# Formulation and Applications of Lipid-Based Nanovehicles: Spotlight on Self-emulsifying Systems

**DOI:** 10.34172/apb.2021.006

**Published:** 2020-11-07

**Authors:** Mohammed M. Mehanna, Amina Tarek Mneimneh

**Affiliations:** Department of Pharmaceutical Technology, Faculty of Pharmacy, Beirut Arab University, Beirut, Lebanon.

**Keywords:** Drug delivery, Lipids, Solubility, Self-nanoemulsifying system

## Abstract

The drug delivery investigation field is continuously widened and adapted to overcome many factors such as poor drug solubility, absorption, rapid metabolism, the variability of drug plasma levels, cellular efflux and many others. Due to resemblance to body constituents and their biocompatibility, lipids offer a promising scheme for poorly water-soluble and lipophilic drugs. Various nanoparticles including vesicular systems, lipid particulate systems, and emulsion systems provide some unique benefits as pharmaceutical carriers in drug and biomolecules delivery systems. Nowadays synthesis is directed toward simple, costless techniques, therefore, self-emulsifying systems have gained superiority over the other carriers. Self nano-emulsifying systems composed of oil, surfactant, and co-surfactant emulsified upon contact with an aqueous medium, has been widely exploited. This review attempts to provide a comprehensive interpretation of different types of lipid-based carriers emphasizing on the self-nanoemulsifying system, why it is gaining interest, formulation, composition, and applications.

## Introduction

Synthesizing new drugs alone is not sufficient to establish advancement in drug therapy. The conventional drug delivery systems are destined to failure, due to many factors, mainly low drug solubility, poor absorption, enzymatic degradation, rapid metabolism, cellular efflux and variability in plasma concentration.^1^

Incorporation of lipids in drug delivery has been a trend in the past decades. Lipid-based carriers are composed of phospholipids, cholesterol, cholesterol esters and triglycerides among others.^2^ The physiochemical diversity of lipids, their biocompatibility and their resemblance to body tissue constituents offer a promising system for poorly water-soluble and lipophilic drugs.^3^ Lipid carriers (LCs) provide several advantages that enable it to be an ideal vehicle for drug delivery. Namely; it can be manipulated according to product requirements whether its disease conditions, route of administration, stability, toxicity or efficacy. Besides, lipid-based formulations (LBFs) can provide a controlled release delivery based on their biocompatibility with body tissue after administration, it’s not susceptible to erosion phenomena, the feasibility of scaling up,^4^ moreover, it provides enhanced drug loading, ability to carry both lipophilic and hydrophilic drugs and stability.

However, LCs face certain limitations such as, lipid crystallization that leads to polymorphism with different drug loading capacity, different shapes, and various kinetic distributions. High-pressure homogenization technique is most commonly used and it might cause drug degradation in high molecular weight compounds.

Lipid-based carriers are recognized as safe and efficient hence they have been used as alluring candidates for pharmaceutical, as well as vaccines, diagnostics, and nutraceutical formulations. Therefore, lipid-based drug delivery (LBDD) systems have gained much importance in recent years due to their ability to improve the solubility and bioavailability of drugs with poor water solubility.

Self-emulsifying drug delivery systems which belongs to LBFs are efficient, sophisticated, and more patient compliant formulation method for poorly water soluble drugs. It may enhance drug solubility, dissolution behavior in the GIT, gut permeability and thus may increase the absorption of the poorly water soluble model drug. This paper illustrates different types of LBFs to be precise, emulsions, vesicular systems, and lipid particulate systems and their subcategories, focusing on self-nanoemulsifying systems and their applications in the pharmaceutical field.

## Materials and Methods

In this review, related articles and research papers from different reliable researchers and database such as Elsevier, Springer and MDPI were collected and discussed. The search was constructed based on the following keywords: lipid-based drug delivery, self-nanoemulsifying system, lipid vesicular systems.

## Type of LBDD system

LBDD systems are classified into vesicular systems, lipid particulate systems, and emulsion systems.

### Vesicular systems

#### Liposomes

**Figure 1:**
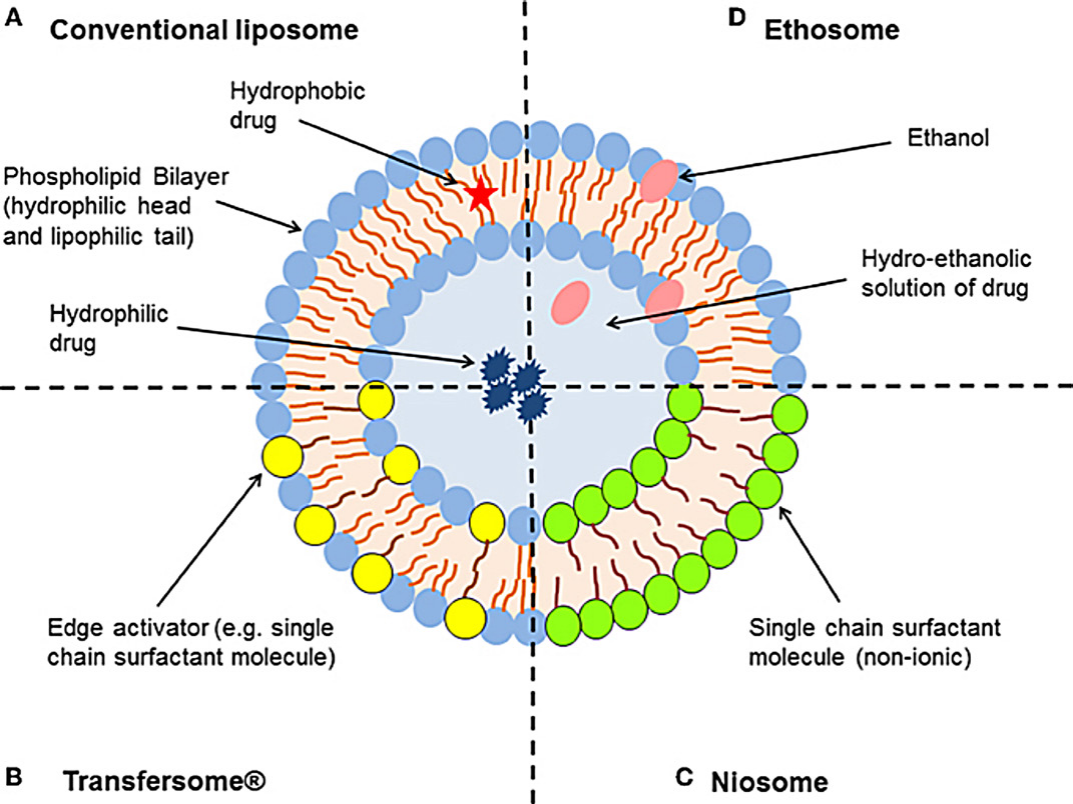
Schematic representation of the different types of lipid-based vesicular delivery systems. (A) Conventional liposomes generally consist of a lipid bilayer composed of phospholipids and cholesterol, which encloses an aqueous core. Liposome characteristics can be modified by the addition of surfactants to form (B) Transfersomes^®^ and (C) niosomes (depending on the ratio of phospholipid to surfactant), or relatively high concentrations of ethanol to form (D) ethosomes. Modified from Hua.^33^

Liposomes are microscopic, colloidal, concentric bilayered vesicles (Figure 1A) with diameter that ranges from 0.02 to 10 μm,^5^ constituted mainly of amphiphilic phospholipids.^6^ Upon contact with an aqueous medium, they assemble as a complex to shield their hydrophobic parts. Stealth porphyrin-phospholipid liposomes with balanced lipid ratios, has been established to prolong the blood circulation time of doxorubicin (Dox). The half-life of Dox in mice was 21.9h and stable for months. Following intravenous injection, Dox deposition increased by 7-folds subcutaneously.^7^ This type of liposome was able to accomplish both rapid light induced release rate and high storage and serum stability with long blood circulation. Many liposomal preparations are in phase Ⅰ or Ⅱ clinical trials such as annamycin-loaded liposomes for treating breast cancer and acute lymphocytic leukemia.^8^ Liposomal drug formulations are also available for intravenous and intramuscular applications. For example, Exparel^®^(2011) is a bupivacaine intravenous used for pain management, and Marqibo^®^(2012) is a vincristine used for acute lymphoblastic leukaemia.^9^ On September 2018, the FDA approved a new drug, Arikayce (amikacin liposome inhalation suspension), for the treatment of lung disease caused by *Mycobacterium avium* complex in patients who do not respond to conventional treatment.^10^

#### Niosomes

In the structure and function, niosomes resemble liposomes. They are minute multi-lamellar formulated by the addition of non-ionic surfactant to cholesterol with successive hydration in aqueous media (Figure 1C). Niosomes can overcome liposomes drawbacks such as chemical instability, purity of phospholipids and high cost, also niosomes provide high penetration ability. Jyouti et al prepared inhalable curcumin loaded freeze-dried cationic small unilamellar niosomes by a reverse-phase evaporation method. The cationic niosomes showed higher release 94%, with long term stability. The freeze-dried cationic niosomes inhibited the A549 lung cancer cells proliferation at the IC50 of 3.1 μM, significantly lower than 7.5 μM of optimized freeze-dried niosomes and curcumin suspension 32 μM, this formulation succeeded in overcoming the poor physiochemical and biocompatibility problems of delivering curcumin to cancerous lung cells.^11^

#### Pharmacosomes

Pharmacosomes are colloidal vesicles, micelles or hexagonal assemblies that are attached covalently to phospholipid with a nanometric size. They can incorporate both hydrophilic and lipophilic drugs, with high entrapment efficiency, target delivery to the site of action, increase bioavailability of poorly soluble drugs, reduce cost, adverse effects and toxicity.^12^ Semalty et al developed pharmacosomes of aceclofenac with higher drug content 91.88% (w/w). The prepared pharmacosome showed higher solubility than aceclofenac alone, with increased drug release over 4 hours during dissolution study.^13^

#### Phytosomes / Herbosomes

Phytosomes are cell-like design; amphiphilic and that what helps in increasing the bioavailability of active phytochemical constituents as they can easily permeate and cross the lipid membrane. These systems have gained a lot of interest lately. Boswellic acid uses have been limited for its low bioavailability and high first pass hepatic metabolism. Sahu et al formulated boswellic acid loaded phytosomes. The preparation showed a high sustained release (80%) for 8hrs, indicating rapid penetration through the skin may be because of nanosized vesicular size The entrapment efficiency increased to 74% with the increase in concentration of cholesterol and ethanol.^14^

#### Transfersomes / Penetrosomes

Transfersomes or elastic liposomes (Figure 1B); these vesicular systems are a type of manipulated liposomes that are ultra-deformable due to the presence of edge activator or surfactants, thus able to deliver the drug into or through the skin to reach systemic circulation with high entrapment efficiency. Mehanna et al developed penetrosomes for the transdermal delivery of tadalafil. The deformability of penetrosomes provided a potential delivery of tadalafil to avoid its oral administration side effects.^15^

#### Ethosomes

Ethosomes are ethanolic liposomes (Figure 1D) that act as a non-invasive carrier system to deliver biologically active agents (hydrophilic and lipophilic) to deeper layers of the skin and systemic circulation. The presence of ethanol provides high stability and disrupts the skin lipid bilayer to enhance skin penetration. These ethanolic vesicles can incorporate as well as amphiphilic molecules.^16^ Bodade et al showed that ethosomal system was able to enhance the entrapment efficacy of repaglinide (75% to 92%). The ex-vivo skin permeation test revealed higher permeation (64%–97%) through excised rat skin when compared to free drug for treatment of diabetes with a sustained release behavior (69% over 24h) and thus reducing dose frequency.^17^

#### Aquasomes

Aquasomes are self-assembled ceramic nanostructures; they consist of three layers. A solid nanocrystalline core made up of polymers (albumin, acrylate or gelatin) or ceramic (diamond or calcium phosphate), coated with carbohydrate film on which active molecules are adhered to, in addition to tin oxide layer for structural stability.^18^ The main application for aquasomes is delivering vaccines, insulin, red blood cells substitute for hemoglobin, enzymes and gene therapy.^19^

#### Novasomes

Novasomes are modified types of liposomes with a diameter ranging from 0.1 to 1 μm. It is a mixture of polyoxyethylene fatty acids, free fatty acids, and cholesterol, forming 2-7 bilayers surrounding an amphipathic core. Novasome surface charge can be neutral, negative or positive.^20^ Abd-Elal et al prepared intranasal zolmitriptan novasome that showed a significant increase in brain targeting (99%) compared to the intravenous administration for migraine attacks.^21^

#### Vesosomes

Vesosomes are a multi-compartment framework with separate inner compartments withdrawn from the external membrane. Each can incorporate varied materials and have a different composition. Due to their multi-structure, they protect the encapsulated contents and demonstrates extended-release of drug.^6^

#### Colloidosomes

Colloidosomes are microcapsules whose shells are composed of colloidal particles.^22^ Microencapsulation enables the controlled release of active ingredients, thus colloidosomes are the choice of systems for encapsulation and controlled drug release.^23^ Nan et al prepared chitosan-coated alginate monodisperse colloidosomes for oral delivery of insulin with high drug encapsulation efficiency (up to 96.7%) and an obvious pH-sensitive release profile.^24^

### Lipid particulate systems

These nanoparticles are formulated from solid or a mixture between solid and liquid lipids and emulsifiers. Lipid nanoparticulate systems have advantages over other systems specifically; ease of scaling up, the biodegradable materials used, low toxicity, drug solubility enhancement and the possibility of combining hydrophilic and lipophilic drug.^25^ All of those explain the increased interest in the field of lipid nanoparticles in the different routes of administration including peroral, dermal, parenteral, pulmonary, in addition to nasal, ocular and cerebral applications.^26-29^

#### Lipospheres

Lipospheres or lipid microspheres are encapsulating systems with a diameter range between 0.1-100 μm. They consist of hydrophobic solid triglyceride fat core stabilized by phospholipids on its surface, and an innermost core holding the therapeutic agent and dispersed in a lipid matrix. Lipospheres are widely used for parenteral delivery as they possess stability over a while at room temperature with no particular undesirable effects, even at high dose levels. These are employed for the controlled delivery of several types of drugs including local anesthetics, anti-inflammatory compounds, anticancer, vaccines, and antibiotics agents.^30^ Lipospheres can be prepared by a solvent evaporation method, melt dispersion technique, sonication technique and evaporation method.^31^ Nasr et al proved that lipospheres can be a promising tool for topical delivery of aceclofenac possessing superior anti-inflammatory effects compared with the marketed product along with high stability and drug entrapment.^32^

#### Solid lipid nanoparticles

**Figure 2:**
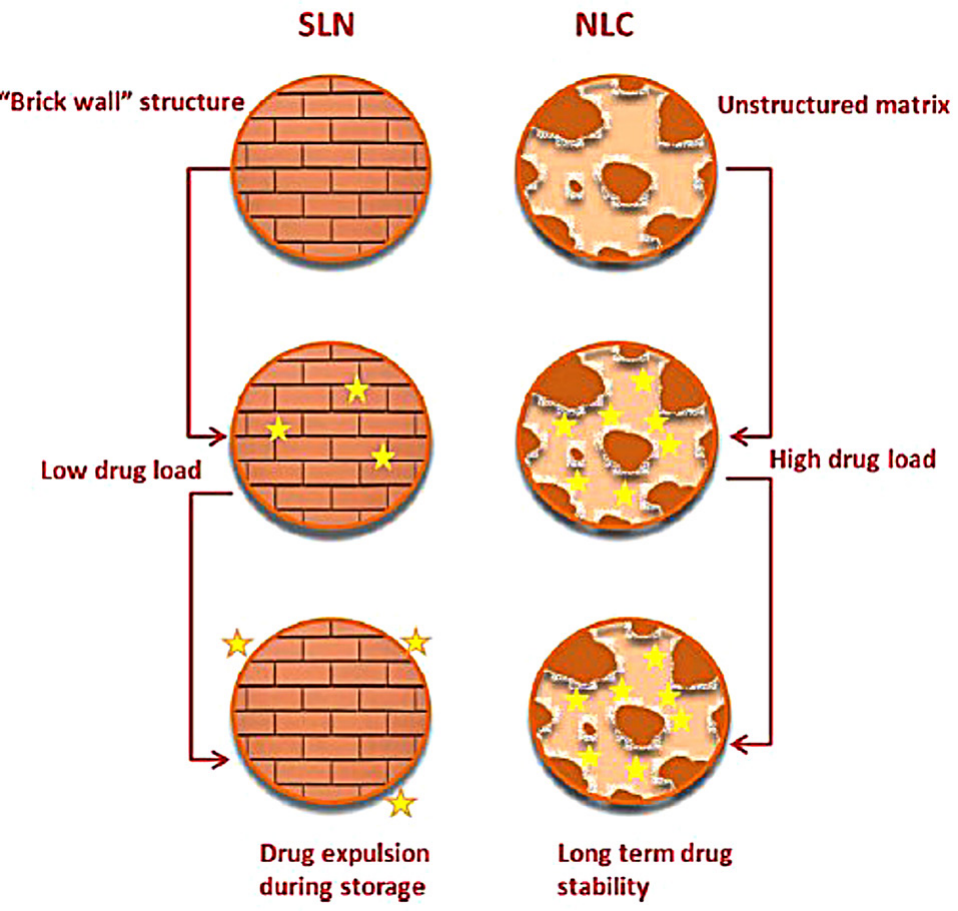
Schematic illustration of SLN structure versus NLC structure, where NLC advantages over SLNs are highlighted. Reproduced from Ref.39

Solid lipid nanoparticles (SLNs) are the first type of lipid nanoparticles reported for drug delivery. SLNs were developed in the middle of the 1990s as an alternative for liposomes, emulsions, and polymeric nanoparticles. This colloidal delivery system is composed of biocompatible lipid nucleus and an amphiphilic surfactant outer shell with a size of 50 to 1000 nm (Figure 2). Depending on drug thermal stability, it is incorporated in the solid lipid colloidal system either by cold or hot homogenization techniques.^34^ The advantages of SLN systems over others are compromised by their nano-size range which offers a narrow window of distribution required for targeted site delivery, protection for the incorporated drug from chemical degradation, organic solvents free and thus ease of industrial scaling up.^35^ Commonly used methods for the preparation of SLNs are high-pressure homogenization, solvent emulsification, evaporation or diffusion, supercritical fluid, ultrasonication or high-speed homogenization and spray drying.^36^ Kelidari et al formulated voriconazole-loaded SLNs by probe ultrasonication technique. His study showed for the first time that this system can be employed as an effective delivery system for voriconazole against azole-resistant *Aspergillosis fumigatus* isolates, as SLNs increased its dissolution and bioavailability.^37^ SLNs drug release is characterized by three patterns; homogeneous release when the melting points of drug and lipid are in steady-state; lipid-enriched core when lipids melt at higher temperature than drug and drug encapsulated core when lipid liquefies at point earlier than drug. Some disadvantages face SLNs include particle size growing and imperfection in solid core upon drug loading, variable gelation tendency, drug escape during storage and low incorporation due to crystalline SLNs core.^38^

#### Nanostructure lipid carriers

**Figure 3:**
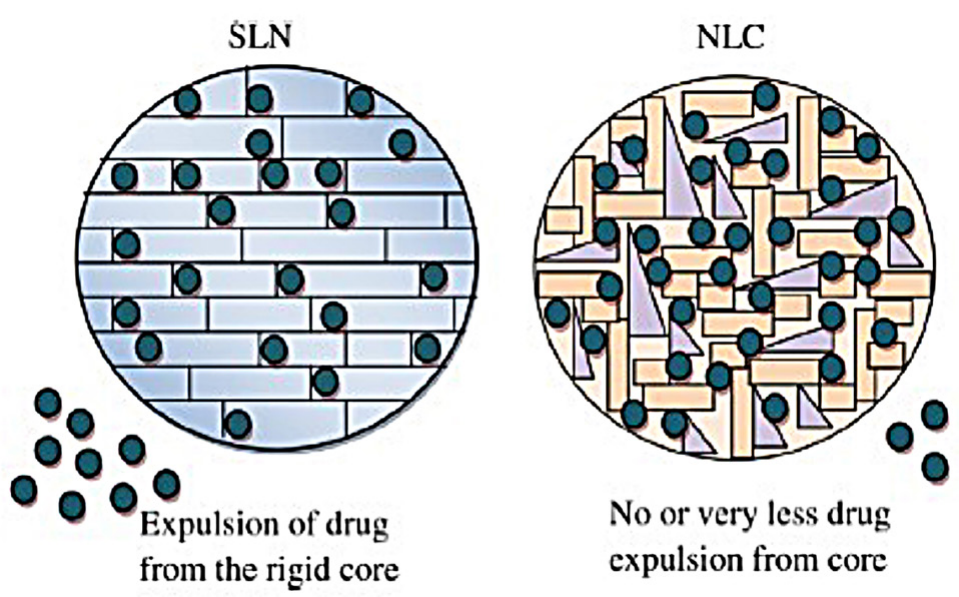
Illustration of stability of SLN and NLC during storage. Reproduced with permission from Salvi and Pawar.^43^

The second generation of lipid carriers is nanostructured lipid carriers (NLCs), which are unstructured solid lipid matrix prepared by blending solid and liquid lipids with an aqueous phase along with surfactant or mixture of surfactants (Figure 2). Among many preparation techniques, a high-pressure homogenization process is the preferred one as no solvent is needed. The hot surfactant solution is added to the hot blended and melted lipids with the drug, the resulted microemulsion is homogenized under high pressure to yield hot nanoemulsion. The nanoemulsion is cooled and NLC is formed. This process can be easily scaled up.^40^ NLCs have three types, imperfect type, amorphous type, and multiple types. The use of generally recognized as safe (GRAS) materials, the large-scalable production, and improved drug safety allow NLCs to be an attractive delivery system candidate for the pharmaceutical market. Meloxicam NLC gel was prepared by Khurana et al and showed sustained release pattern, enhanced skin permeation and a better deposition into the dermis in comparison with control meloxicam gel.^41^ Fathi et al prepared oral simvastatin NLCs that showed improved and prolonged reduction in the total cholesterol and non-high density lipoprotein compared to drug suspension with 4-folds increase in its bioavailability.^42^ During storage, SLN and NLC act differently (Figure 3). SLN is formulated completely from solid lipids; therefore, after crystallization, it forms a rigid core restricting the movement of drugs within the core, leading to the expulsion of the drug into dispersion media. Due to this phenomenon, entrapment efficiency is lowered. However, the composition of NLCs is the mixture of solid and liquid lipids, thus an imperfect core is formed. Such core provides higher drug loading and enough space for the incorporation of the drug. Hence during storage, the drug is not expelled out of the core.^43^

#### Lipid-drug conjugates

Lipid-drug conjugates are drug molecules that have been covalently modified with lipids. According to the type of drug and the lipid used different conjugation strategies are utilized including; conjugation with lipids and chemical bonding. Lipid-drug conjugates offer several advantages, including specific site targeting, low toxicity, and better drug loading into delivery systems, enhanced oral bioavailability, and tumor targeting. Pharmacokinetics study showed that linking paclitaxel to docosahexaenoic acid provides a sustained high drug concentration in plasma for a longer time compared with paclitaxel alone, thus increasing dose by 4.4-folds with no increase in toxicity.^44^

### Emulsions

Emulsions are liquid-liquid heterogeneous colloidal systems composed of at least two immiscible liquids, water, and oil that are uniformly dispersed as fine droplets by mechanical agitation. Emulsions are usually classified as oil-in-water (O/W) or water-in-oil (W/O) depending on the nature of the continuous phase. They are thermodynamically unstable, yet they can be stabilized by an emulsifying agent mainly surfactants.^45^

#### Microemulsions

Microemulsions are clear, thermodynamically stable isotropic mixture of oil, water, surfactant, and co-surfactant, which were first introduced by Hoar and Schulman in 1940s.^46^ Microemulsions show several interesting characteristics including enhancing drug solubility, thermodynamic stability, ease of preparation and scaling up, and high drug loading capacity.^47^ Microemulsions are widely formulated in the field of cosmetics and cosmeceutical, especially for skin and hair preparations. Clobetasol propionate-loaded microemulsion based gel prepared by Patel et al showed that higher drug permeation into the skin microemulsion (60.33 ± 4.67%) compared with the marketed product (37.77 ± 0.77%) with better retention in the skin and minimal irritation potential thus proved to be a promising formulation for the effective treatment of vitiligo.^48^

#### Pickering emulsions

Pickering emulsions are simple emulsions that depend on lipid having inner nanostructures stabilized by silica, clays, calcium carbonate or titanium dioxide. This system can be any type of emulsions, oil/water (o/w), water in oil (w/o) or even multiple emulsions stabilized by solid particles rather than surfactant.^6^ The main advantage of this system is its resistance to coalescence due to the stability provided by solid particles. Shah et al prepared chitosan-tripolyphosphate nanoparticles stabilized Pickering emulsion to deliver curcumin. The stability of curcumin in the Pickering emulsion was significantly improved with a sustained release profile over a long period.^49^

#### Nanoemulsions

Nanoemulsions (NE), oil-in-water (o/w) or water-in-oil (w/o), are heterogeneous dispersion with droplet size in the nanometric range between 20-200 nm. Due to this small size, they appear transparent or slightly turbid and have good stability against flocculation, coalescence or phase separation.^50^ There are many structural similarities between nanoemulsion and microemulsion, however, there are also some vital differences represented in Table 1 and there has been great misperception about the exact nature and classifications of these different systems.^51^

**Table 1:** Differences between nanoemulsions and microemulsions^51,52^

**Characteristics**	**Nanoemulsions**	**Microemulsions**
Definition	Colloidal system of two immiscible liquids	Swollen micelle
Order of mixing	Only formed when surfactants are first mixed with oils	Order of mixing does not matter
Thermodynamic stability	Non-equilibrium	Equilibrium
Kinetic stability	Stable	Less stable
Size	<200 nm	10-100 μm
Droplet size control	Depend on surfactant/oil ratio	Exhibit different phases with different nanosize morphology
Destabilization	Ostwald ripening only	Dilution and temperature change
Preparation	High energy or low energy methods	Spontaneous emulsification (depends upon temperature, composition, and pressure)
Characterization methods with dilution step	Applicable	Invalid as the droplet size increase
Dilution and temperature	Robust	Strongly affected

The advantages of NE have appealed scientists to explore them in various fields especially the pharmaceutical drug-delivering area. Whether O/W or W/O, nanoemulsions can solubilize both hydrophilic and hydrophobic drugs and thus enhance their oral bioavailability such as curcumin, ramipril and ezetimibe.^53-55^ Nanoemulsions have long term stability and hence provide long shelf-life of the formulated agents. Encapsulation in nanoemulsion protects therapeutic agents against enzymatic and chemical factors. They are biodegradable, biocompatible and easy to prepare. The rapid skin penetration, low viscosity and the almost translucent nature of the nanoemulsion provide a visual appeal and patient compliance especially with dermal, ‘roll-on’-type formulations, sprays, and gels.^56^

**Figure 4:**
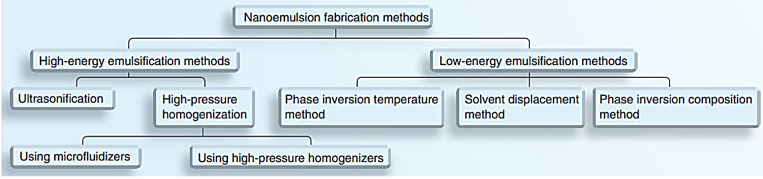
Various methods for nanoemulsion fabrication. Reproduced with permission from Date et al.^58^

Fabricating methods used for nanoemulsion preparation is divided into high energy and low energy emulsification methods^57^ as illustrated in Figure 4.

## High energy methods

### High-pressure homogenization

High-pressure homogenization is the widely used technique in the preparation of nanoemulsion. This method can be used on a large scale; however, a high amount of energy and the evolved temperature during emulsification are not suitable for thermosensitive therapeutic agents. It provides high pressure up to 20000 psi that produces nanoemulsion in very small particle size (up to 1 nm), yet the small size of the nanoemulsion depends on the number of cycles, as the number of homogenization cycles increases the smaller the droplet size is produced. The major drawbacks of high-pressure homogenization are poor productivity and product deterioration.^59^

### Microfluidization

Microfluidization shares the same principle of high-pressure homogenizer, yet the difference is utilizing microchannels to produce the droplets. In this technique, the cycle is repeated until the desired size is achieved; sometimes nitrogen atmosphere is used to filter large droplets.^57^ The advantages of this method include smaller droplet, higher efficiency of droplet disruption and more uniform droplet distribution. However, microfluidization is expensive and not reproducible on a large scale.^60^

### Ultrasonication 

This method produces kinetically stable nanoemulsions. Sound waves more than 20 kHz are applied and a sonicator probe is introduced into the dispersion of liquids with surfactant and co-surfactant to create mechanical vibration and cavitation, providing high energy to produce small-sized droplets.^61^ Ultrasonication is used on a small scale so it’s not suitable for large volume and care must be taken to prevent coalescence.

## Low- energy methods

### Phase inversion temperature 

Phase inversion temperature (PIT) depends on the ability of polyoxyethylene-type surfactant (nonionic) to change their hydrophilic nature into lipophilic ones depending on the temperature.^61^ This method was first introduced by Shinoda.^62^ If the emulsion prepared at a temperature near phase inversion temperature then rapidly cooled or heated, a small size nanoemulsion with narrow size distribution and kinetically stable one will be formed. If w/o emulsion is rapidly cooled, it converts to o/w, on the opposites if o/w emulsion is rapidly heated, it converts to w/o emulsion.^63^ The advantage of this method is its low cost yet it is only limited to non-ionic surfactants.

### Phase inversion composition

This method is similar to phase inversion temperature, but the difference is that can be achieved by changing the oil to water ratio or surfactant properties. Maestro et al used this method to convert water/oleylammonium chloride–oleylamine–C_12_E_10_/hexadecane into o/w nanoemulsion by introducing a direct cubic liquid crystal phase along the emulsification path allowing the incorporation of the oil in the cubic phase easing its emulsification.^64^ Phase inversion composition method is simple, low cost and doesn’t require organic solvent.^65^

### Solvent diffusion or spontaneous emulsification 

The solvent diffusion method depends on spontaneous emulsification without the need for special equipment. It is achieved by simply mixing oil first into surfactant and injected into the aqueous phase at a fixed temperature.^66^ The spontaneity of the emulsification process depends on optimizing certain parameters namely; the composition and volume of the aqueous and organic phase, temperature, pH, ionic strength, in addition to the mixing conditions such as stirring speed, rate and order of addition.^67^ The main disadvantage of this method is its limitation to small volumes of surfactant and oil and the utilization of organic solvents in some cases.

#### Applications of nanoemulsion

Nanoemulsions have been used in most routes of drug administration, namely, topical, ocular, intranasal, intravenous and oral delivery.

*Intranasal based* nanoemulsion was prepared by Mahajan et al where the optimized nanoemulsion showed a high percentage of drug targeting efficiency (2919.261 ± 5.68) and nose-to-brain drug direct transport percentage (96.574% ± 0.76) proving that this system is a good carrier for saquinavir mesylate to CNS through intranasal route.^68^

*Topical nanoemulsion* prepared by Oliviera et al for the treatment of dermatoses. The *ex-vivo* permeation study showed that 8.5% of the applied 8-methoxy psoralen dose permeated through the biological membranes, and retention in viable skin induced by the NE was almost two-fold higher than a compounded cream (5.04 ± 0.30 μg cm^−2^). These results proposed that the developed nanoemulsion is a promising alternate for 8-methoxy psoralen topical therapy.^69^

*Thermosensitive in-situ ocular* diclofenac*nanogel* prepared byChauhan and Batra as a good replacement for conventional eye drops due to higher permeation and prolonged precorneal residence time. The formulated nanoemulsion *in-situ* gel showed drug release for a longer duration of time (8 h) as compared to the marketed eye drops (3 h), thus sustained drug delivery was achieved, and also the developed formulation exhibited higher permeation across goat cornea in 4 hours. Hence, nanoemulsion was found to be.^70^

*Vehicle in cancer chemotherapy* due to their ability to prolong rate of drug release after intramuscular and intratumoral injection and enhance the transport of anticancer agents via lymphatic system.^71^ Piplartine was formulated into nanoemulsion by Fofaria et al and did not exhibit any toxicity upon administration for 60 days with 1.5-fold increase in oral bioavailability as compared to free piplartine and clear anti-tumor activity at a dose of 10 mg/kg in melanoma tumor-bearing mice.^72^

*Cosmetics;* the active constituents of NE are easily absorbed due to the small size of the droplet and can reduce the water loss from the skin, providing an elegant and stable product that can be formulated as moisturizers and creams. *Opuntia ficus -indica* (O/W) nanoemulsion presented suitable stability for at least 60 days and was able to increase the water content of the stratum corneum for 5 h after application showing its moisturizing efficacy.^73^

### Self-nanoemulsifying drug delivery system (SNEDDS)

SNEDDS is an isotropic mixture of oil, surfactant and co-surfactant emulsified with mild agitation with the therapeutic agent. When diluted with aqueous media with mild stirring, it instantly produces oil-in-water micro or nanoemulsion.^74^ Upon mild agitation followed by dilution with aqueous media, these systems can spontaneously form fine (oil in water) emulsion with a globule size less than 200 nm.^61^ These small droplets containing the dissolved drug in the oil phase have enhanced surface area and thus faster digestion and absorption in the gastrointestinal tract.^75^ The increase in the surface area provides better drug solubility and permeation. The drug can be formulated in a dose less than 25mg and up to 2 g.^76^

#### Composition of self-nanoemulsifying drug delivery system

*Lipid* is an important ingredient in SNEDDS formulation. Modified long and medium-chain triglyceride oils with varying saturation degrees have been used widely for the design of SNEDDS; when used with surfactants these semisynthetic derivatives form good emulsification systems for oral administration.^77^ Lipids not only solubilize a large number of lipophilic drugs but also enhance the drug transport via the intestinal lymphatic system increasing its absorption from the GIT. Thomas *et al.* formulated two SNEDDS with medium-chain (MC) and long-chain (LC) lipid, and found that MC-SNEEDs can incorporate more drug than that of LC-SNEDDS.^78^ *Surfactant* is crucial for the emulsification of the SNEDDS, for achieving high emulsifying performance, the HLB of the surfactant used, their cloud point, viscosity and solubility in the oil phase affect formulation of SNEDDS, the nanoemulsion region and droplet size.^58^ The formulation of effective SNEDDS requires high concentration of surfactants, and therefore the incorporation of the *co-emulsifiers, co-surfactants* or solubilizers in SNEDDS modulate self-nanoemulsification, expand the self-nanoemulsification region, increase drug loading, and droplet size of nanoemulsion.^58^

#### Advantages of SNEDDS

SNEDDS provides long term stability due to the absence of water, orally SNEDDS suffer from no palatability problems as they can be filled into capsules or formulated into tablets.^58^ The success of any drug delivery system depends on its industrial applicability, ease of manufacture, scale-up, and transformation from research to the market. Due to high surfactant/co-surfactant to oil ratios, SNEDDS have more drug-loading capacity which is the success of this system. Since the rapid onset of action is required in many pathological cases, such as hypertension, angina, and inflammation, SNEDDS enhances the oral absorption of the drug and thus provides fast onset of action.

#### Spontaneous emulsification process

The mechanism of spontaneous emulsification is not fully understood, as it can occur through different mechanisms. According to what described by Reiss, self-emulsification takes place when the entropy change that favors dispersion is greater than the energy required to increase the surface area of the dispersion.^79^ In the conventional emulsion systems, surfactants decrease the interfacial energy by creating a layer around the internal phase of globules acting as a barrier against coalescence but still, these emulsions are thermodynamically unstable. In a spontaneous self-emulsifying system, the free energy needed to form the emulsion is either very low or negative. Groves and Galindez reported that the liquid crystalline phase formed between the oil/surfactant and water phases effectively swells allowing spontaneous formation of an interface between the oil droplets and water.^80^

#### Application of self-nanoemulsifying drug delivery system

The strength of SNEDDS is not limited to augment the dissolution rate only it is extended to overcome mucus gel barrier, delivery of biomolecules, and even drug targeting.^81^

#### Solubility and bioavailability improvement

When the drug is incorporated into SNEDDS, it will be solubilized at the site of absorption, making it easy to pass the biological membrane and reach the site of action, hence the bioavailability problem of the drug is bypassed.

Joshi et al formulated SNEDS of curcumin that showed improvement in C_max_ and AUC_(0-t)_ by 1632.1% and 7411.1%, respectively compared to an aqueous suspension of free curcumin, with better results against diabetic neuropathy.^82^ Glipizide is an oral antidiabetic drug. Chemically is a weak acid with poor water solubility. Glipizide solid SNEDS has been formulated by Dash et al where the optimized formula showed an enhancement in solubility and dissolution.^83^ Shakeel et al also formulated self-nanoemulsifying drug delivery system of indomethacin to improve its solubility as well as *in-vitro* dissolution rate, the solubility study results showed 4573 folds increase in solubility, and drug release was faster with 93% of the drug was released in first 15 min of study as compared to 48% from commercial capsules.^84^ Researches have shown that SNEDDS can be achieved in different formulations without compromising bioavailability (Table 2).

**Table 2:** Summary of some research articles describing different SNEDDS formulations

**Drug**	**Excipients**	**Formulation**	**Bioavailability (in-vitro/in-vivo results)**	**Reference**
Cyclosporine A	Labrafil^®^ Transcutol^®^, Cremophor^®^	Controlled release osmotic pump tablets	80% drug release in 12 h	^85^
Ziprasidone	Capmul^®^ Labrasol^®^, PEG 400*	Sustained release pellets	95% drug release within 12 h	^86^
Embelin	Capryol^®^, Acrosyl^®^,PEG 400*	Tablets	96.5% drug released within 15 min *in-vitro* compared to 5% pure drug	^87^
Glimepiride	Miglyol^®^, Tween^®^, PEG 400*	S-SNEDDS powder-filled hard gelatin capsules	95% *in-vitro* drug release within 1 h	^88^
Valsartan	Capmul^®^, Labrasol^®^, Tween^®^	Tablets	3–3.5-Fold increased dissolution rate with almost all drug released within 1 h	^89^

*Polyethylene glycol (PEG 400)

#### Mucus permeation enhancer

Mucus barriers are present in buccal, ocular and nasal cavities, also in intestine, lung, and vagina. Secretion and clearance rates of mucus are fast, so the mucus barrier creates a challenge for drug carriers to reach the epithelial surface and remains there for the required time. Due to the hydrophobic surface of the nanodroplets of SNEDDS, the interaction with the mucus barrier is minimal and enables it to pass without being entrapped.^90^ Current treatments for melanoma and psoriasis are inefficient due to poor transcutaneous permeation, thus creating a need for a new colloidal carrier. Pund et al prepared leflunomide nanoemulgel for the localized treatment of psoriatic as well as melanoma. The *ex-vivo* permeation study showed a significant enhancement in the flux of 5.65 times with nanoemulgel formulation compared to ordinary gel, permeability coefficient increased from 5.93 to 33.48 cm^-2^ min-^1^ and drug deposition in the skin from 222.7 to 1287.2 μg.^91^ Bifonazole-loaded self-emulsifying system was formulated by Alhakamy and Hosny utilizing Peceol^®^, Kolliphore^®^EL and Plurol Oleique^®^497 for the topical delivery of this antifungal agent, the nano size of the formula enhanced drug antifungal activity and its permeability by 1.85 and 2.179 folds compared to aqueous suspension, due to the tendency of Kolliphore^®^EL toward the cellular membrane and the formation of micelles by Plurol Oleique^®^497, thereby, extracting the lipids from the skin and enabling greater penetration of bifonazole across skin deeper layer proving that this system is an efficient vehicle for transdermal delivery.^92^

### Bio-molecules delivery

Bio-molecules (lipids, proteins, genes, and polysaccharides) have earned great attention as modern therapeutics due to their high selectivity, specificity, and low-toxic effects. Yet they suffer from low bioavailability due to poor permeation because of their large size and hydrophilicity (proteins) and enzymatic degradation.^93^

Polypeptide-k (PPK) is a peptide extracted from dried ripened seeds of *Momordica charantia* that has been reported for its antidiabetic activity by inhibiting α-glucosidase and α-amylase, yet its oral delivery is still a challenge due to limited dissolution, bioavailability along with the enzymatic degradation in the GIT.^94^ Self-emulsifying delivery system of PPK was stable against pH change, dilution and temperature changes, with enhanced dissolution profile and a potentiated antidiabetic activity.^94^ Gene therapy requires the availability of genetic material at the targeted site. Gene therapy is a promising tool for the progression of many diseases such as cancer, AIDS, Parkinson’s and Alzheimer’s. The challenge with delivering non-viral genes is due to their poor cellular uptake and enzymatic degradation of the DNA-based drugs especially in the oral route. DNA was incorporated in the lipid phase of the nanoemulsion, creating a protective effect against degradation via DNase I enzyme. It was also reported that the incorporation into the lipid phase and the hydrophobic ion pairing didn’t decrease the uptake or the transfection efficiency compared with marketed liposomal transfection reagent.^93^ Vitamin K is one of the most important factors needed for the coagulation process, yet it is poorly soluble in water and its absorption is varied and so its bioavailability. Its parenteral administration is accompanied by drawbacks such as extravasation of drug or blood, catheter infections, and thrombosis, so the solution is supplying vitamin K orally; however, its oral administration is limited due to low bioavailability, low solubility, permeability, and its rapid metabolism. Vitamin K self-nanoemulsifying drug delivery system was prepared and was loaded on porous carriers and formulated as lyophilized tablets. Self-nanoemulsifying lyophilized tablets improved both rate and extent of vitamin K absorption, release of vitamin K from the developed formula showed significant superiority compared with the commercial tablets (99% with 60 min) as well as its bioavailability (169.67%). Self-nanoemulsifying lyophilized tablets have a strong influence on the efficiency of vitamin K in the prophylaxis and treatment of bleeding disorders in patients with hepatic dysfunction.^95^

## Conclusion and future perspective

LBDDSs are resourceful carriers that are favored due to their biocompatibility, the flexibility of pharmaceutical lipid excipients, and their compatibility with different dosage forms. With the rise of low-energy and self-emulsification methods, nanoemulsions and SNEDDS regained great attention. The field of SNEDDSs has spread beyond overcoming dissolution and solubility issues, to deliver biomolecules, vaccines, insulin, and mucus membrane permeation, in addition to targeting specific organs. In the future, further tests should be established and exploited for better estimation and assessment of the in-vivo aspect of these systems, and to emphasize the role of each component.

## Conflictof Interest

The authors report no conflict of interest in this work

## Ethical Issues

Not applicable.
